# Supplementary material for: Dynamics of Lipid Profile in Antiretroviral-Naïve HIV-Infected Patients, Treated with TAF-Based Regimens: A Multicenter Observational Study
Source: Biomedicines. 2022 Dec 7;10(12):3164. doi: 10.3390/biomedicines10123164 (PMC9775227; doi:10.3390/biomedicines10123164)
Supplement: Supplementary file 1 [file biomedicines-10-03164-s001.zip › biomedicines-2012409-supplementary.pdf]

Supplementary Figure S1:

Averages of lipids at different timing of follow-up according to ART regimen

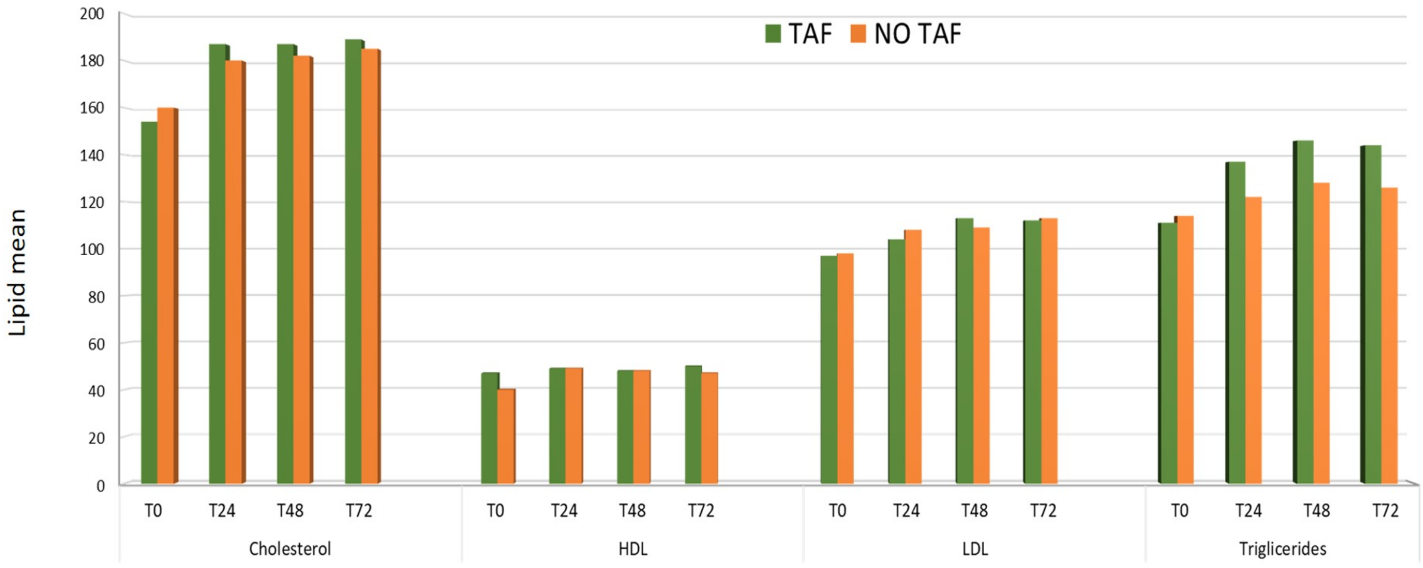

Supplementary Figure S2:

Onset of treatment with statin and hypoglycemic drugs at different weeks of follow-up in Cases and Controls group

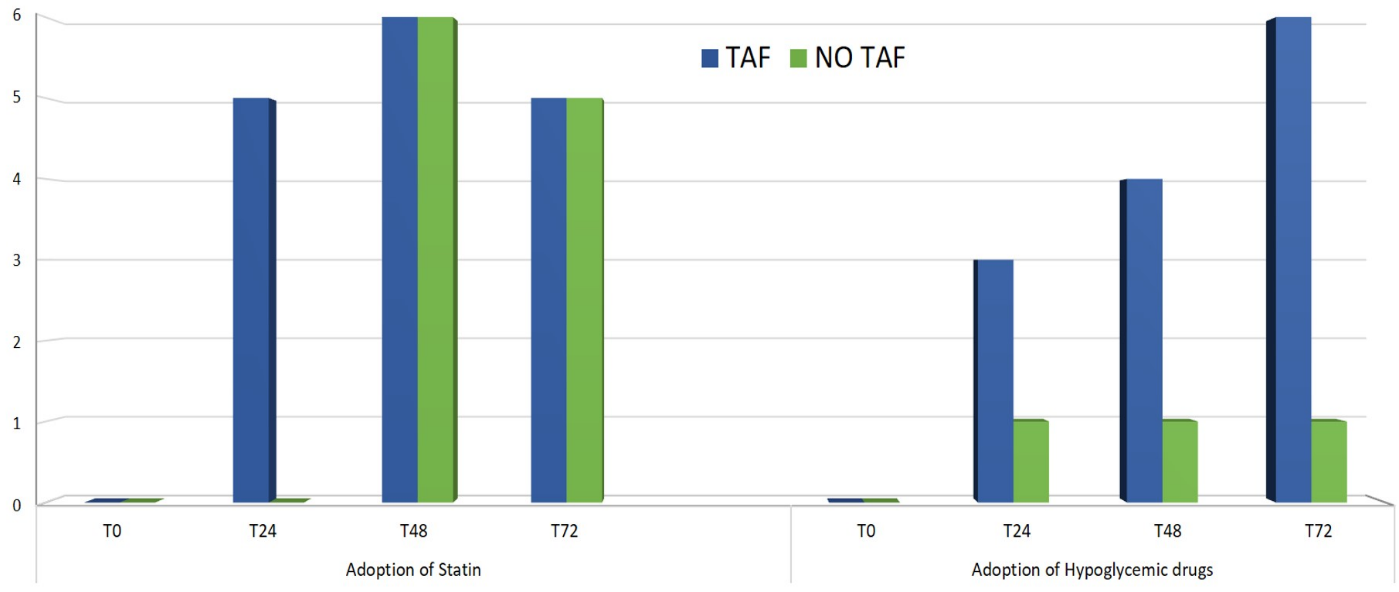

Supplementary Table S1:

Binary logistic regression analysis evaluating the independent factors associated to hypercholesterolemia at different time points, analyzed for different third drug in Cases group

**Binary Logistic Regression**				
Follow-up 24 weeks	Sig.	Exp(B)	95% CI for EXP (B)	
			Inferior	Superior
Age ≥ 50 vs < 50 years old	**0,01**	2,353	1,227	4,510
Sex F vs M	0,144	1,649	0,843	3,225
TAF/FTC/RIL vs NO TAF	0,937	0,961	0,362	2,554
TAF/FTC/EVT/COBI vs NO TAF	0,605	1,188	0,617	2,287
TAF/FTC/DGT vs NO TAF	0,839	0,908	0,358	2,305
Follow-up 48 weeks	Sig.	Exp(B)	95% CI for EXP (B)	
			Inferior	Superior
Age ≥ 50 vs < 50 years old	0,232	1,508	0,769	2,959
Sex F vs M	0,648	1,178	0,582	2,386
TAF/FTC/RIL vs NO TAF	0,998	1,001	0,288	3,476
TAF/FTC/EVT/COBI vs NO TAF	0,164	1,609	0,823	3,145
TAF/FTC/DGT vs NO TAF	0,444	1,418	0,580	3,464
Follow-up 72 weeks	Sig.	Exp(B)	95% CI for EXP (B)	
			Inferior	Superior
Age ≥ 50 vs < 50 years old	0,279	1,468	0,732	2,945
Sex F vs M	0,48	0,479	0,232	0,992
TAF/FTC/RIL vs NO TAF	0,631	1,303	0,443	3,834
TAF/FTC/EVT/COBI vs NO TAF	0,263	1,47	0,749	2,885
TAF/FTC/DGT vs NO TAF	0,855	0,914	0,351	2,381

Supplementary Table S2: Viro-immunological efficacy at different weeks of follow-up in Cases and Controls group

	TAF T0	NO TAF T0	p	TAF T24	NO TAF T24	p	TAF T48	NO TAF T48	p	TAF T72	NO TAF T72	p
CD4+ mean ± SD	401,86 +/- 234,34	354,58 ± 256.69	0,11	568,31 +/- 267,12	583 +/- 329.30	0,68	641,47 +/- 295,57	620,39 +/- 326,83	0,60	709,47 +/- 302,52	699,96 +/- 365,51	0,83
% HIV-RNA <40 copies/ml				124/138 (89,85%)	107/125 (85,6%)	0,38	107/116 (92,24%)	108/118 (91,52%)	0,96	96/104 (92,30%)	110/116 (94,82%)	0,6

	TAF/RIL T0	TAF/EVG/c T0	TAF/INI T0	TAF/RIL T24	TAF/EVG/c T24	TAF/INI T24	TAF/RIL T48	TAF/EVG/c T48	TAF/INI T48	TAF/RIL T72	TAF/EVG/c T72	TAF/INI T72
% HIV-RNA <40 copies/ml				25/29 (86,2%)	71/77 (92,2%)	29/32 (90,6%)	18/19 (94,7%)	62/67 (92,5%)	27/30 (90%)	16/17 (94,1%)	56/60 (93,3%)	24/27 (88,8%)
CD4+ mean ± SD	534,79	377,54	343,27	652,24	555,86	524,36	753,15	589,71	687,89	738,94	692,58	727,37
